# Perceived Well-Being among Adults with Diabetes and Hypertension: A National Study

**DOI:** 10.3390/healthcare12080844

**Published:** 2024-04-16

**Authors:** Leona Yuen-Ling Leung, Hon-Lon Tam, Isaac Sze-Him Leung, Alex Siu-Wing Chan, Yueheng Yin, Xiubin Zhang, Aimei Mao, Pak-Leng Cheong

**Affiliations:** 1 School of Nursing and Health Studies, Hong Kong Metropolitan University, Hong Kong SAR, China; lleung@hkmu.edu.hk; 2 The Nethersole School of Nursing, The Chinese University of Hong Kong, Hong Kong SAR, China; 3 Department of Statistics, The Chinese University of Hong Kong, Hong Kong SAR, China; shleung@cuhk.edu.hk; 4 Department of Applied Social Sciences, The Hong Kong Polytechnic University of Hong Kong, Hong Kong SAR, China; chansw.alex@gmail.com; 5 School of Nursing, Nanjing Medical University, Nanjing 210029, China; yinyueheng@njmu.edu.cn; 6 School of Public Health, National Heart and Lung Institute, Imperial College London, London W12 7RQ, UK; xiubin.zhang@imperial.ac.uk; 7 Department of Education, Kiang Wu Nursing College of Macau, Macau SAR, China; maoaimei@kwnc.edu.mo (A.M.); joecheong@kwnc.edu.mo (P.-L.C.)

**Keywords:** diabetes, hypertension, population surveillance, quality of life

## Abstract

Perceived health and distresses are associated with the practice of lifestyle modifications, which increases the risk of diabetes and hypertension-related complications. This study aimed to define the characteristics and distribution of perceived health and distresses across the states between people with diabetes and hypertension. Data were derived from a national survey of US adults aged ≥18 years who were interviewed via phone call. Perceived health and distresses were assessed through corresponding questions. An amount of 333,316 respondents (43,911 with diabetes and 130,960 with hypertension) were included in the analysis; 61.8% of people with diabetes and 74.5% of people with hypertension reported having good or better health, while residents in the Southwest region perceived poor health statuses and more distresses. Education level (diabetes: odds ratio [OR] = 0.47–0.79, hypertension: OR = 0.42–0.76), employment status level (diabetes: OR = 1.40–2.22, hypertension: OR = 1.56–2.49), and household income (diabetes: OR = 0.22–0.65, hypertension: OR = 0.15–0.78) were significant factors associated with poorly perceived health among people with diabetes and hypertension, and the use of technology and strategies for policymakers are suggested to improve the perceived health status in this regard.

## 1. Introduction

The World Health Organization (WHO) reported that noncommunicable diseases accounted for 74% of deaths worldwide in 2019 [1], while hypertension and diabetes were the first and the third leading risk factors to cause disease burden globally [2]. Hyperten-sion and diabetes are also major risk factors in various health problems, such as heart diseases, stroke, and kidney diseases [1]. In addition, hypertension and diabetes increase the health expenditure worldwide [3,4]. Different types of pharmacological and non-pharmacological interventions have been developed to manage hypertension and diabetes [5,6,7]; however, numerous side effects were reported on the use of pharmacological interventions [8], and so non-pharmacological interventions are highly recommended. Among the non-pharmacological interventions, adopting healthy lifestyles is recommended by the WHO to reduce risk of noncommunicable diseases, and different international organizations provide guidelines for management of hypertension and diabetes to reduce risk of complications [5,6,7,9]. However, a national study of 278,064 individuals found that people with hypertension and diabetes were the least likely to practice healthy lifestyles (odds ratio [OR] = 0.48–0.51) [10], also revealing that people with perceived good or better health were more likely to practice healthy lifestyles (OR = 1.59) [10]. A study of 5123 individuals indicated that people perceived a poor health status when they had physical and mental distress [11]. Another study suggested that perceived health status was related to the practice of healthy lifestyles, physical distress, and mental distress [12].

The mental distress among people with diabetes has been widely discussed in recent years. A systematic review of 55 studies (including 36,998 individuals) found that the prevalence of mental distress among people with diabetes was 36% [13]. A high level of mental distress could lead to poor glycemia control, resulting in an increase in complications and mortality [14]. However, the mental distress among people with hypertension has been less discussed, and the low prevalence (3.2%) might be a possible reason [15]. On the other hand, physical distress could limit people’s daily activity [16], but physical distress among people with diabetes and hypertension has not been extensively investigated. Hence, the findings of this study provide valuable information to develop interventions targeted at improving perceived health and reducing physical and mental distress among people with diabetes and hypertension.

### Aims

This study aimed to (1) define the characteristics of people with diabetes and hyper-tension, (2) illustrate the distribution of perceived health and distresses across the states, and (3) determine the factors associated with perceived health and distresses at a national level.

## 2. Materials and Methods

This cross-sectional study was reported in accordance with the Strengthening the Reporting of Observational Studies in Epidemiology guideline [17]. The data were de-identified and publicly available from the US Centers for Disease Control. No institutional review board approval or patient informed consent was required.

### 2.1. Data Sources

The data from the 2021 Behavioral Risk Factor Surveillance System (BRFSS) survey were used [18]. The survey investigated health-related behaviors and health conditions of noninstitutionalized US adults aged 18 years and above. The surveyor used the random-digit-dialing technique to generate random phone numbers for telephone and mobile phone interviews across all states in the United States. Data collection was conducted throughout the entire year of 2021, while some states continued data collection into early 2022 [18]. Florida was excluded in this annual survey because the data collection time did not fulfil the minimum requirements for inclusion. The median response rate was 44% [18].

### 2.2. Study Population

Cases for which data regarding sociodemographic variables or health conditions were missing and individuals who refused to answer questions were excluded from the study. We included 333,316/438,693 (75.9%) individuals from the 2021 BRFSS survey.

### 2.3. Outcome Variables

#### 2.3.1. Sociodemographic Variables

Following a previous study [10], serval sociodemographic variables were extracted from the BRFSS survey. Employment status was recategorized, while the rest of the variables followed the categorizations from the survey.

#### 2.3.2. Perceived Health and Distresses

Related items from the BRFSS survey were extracted to assess individual perceived health and distress. Individuals selected appropriate responses, including ‘excellent’, ‘very good’, ‘good’, ‘fair’, and ‘poor’, to indicate their perceived health status. The responses were categorized as ‘fair or poor’ (including the rating of fair and poor) and ‘good or better’ (including the ratings of good, very good, and excellent) in regression analysis. Further, perceived distress was assessed by asking individuals about the number of unhealthy days caused by physical distress (related to physical illness and injury) or mental distress (related to stress, depression, and problems with emotions) over the previous 30 days. The number of days of daily activity limitation caused by physical or mental distress was also assessed.

### 2.4. Data Analysis and Visualization

Data were analyzed using the R statistical software, v. 4.3.2 (R Foundation for Statistical Computing). Logistic regression analysis was used to investigate which sociodemographic characteristics (independent variables) were associated with perceived health status and distresses (dependent variables). All statistical tests were two-tailed, and variables were considered significant at a significance level of <0.05.

The distribution of health status and distresses among people with diabetes and hypertension in different states was visualized using the Geographic Heat Map add-in featured in Excel 2019, v. 16. The blue color was for diabetes, while green was for hypertension. The color would be lighter if the condition was better.

## 3. Results

We analyzed 333,316 respondents, and 43,911 (13.2%) and 130,960 (39.3%) were diagnosed with diabetes and hypertension, respectively (Table 1). Over 50% of people with diabetes and hypertension were older adults (aged 65 years and older) and retired or unable to work. Regarding the health status, 83.6% (*n* = 278,783) of individuals were perceived to have good or better health; however, only 61.8% (*n* = 27,142) and 74.5% (*n* = 97,534) of people with diabetes and hypertension were reported to have good or better health. Additionally, people with diabetes and hypertension were reported to have more distresses, and people with diabetes reported 7.25 ± 11.05 days and 4.79 ± 9.05 days of physical distress and mental distress in a month, respectively. These distresses could limit their daily activity by 7.89 ± 10.96 days in a month. People with hypertension also reported more distresses than the general population, but the level was less severe than people with diabetes (Table 1).

### 3.1. Perceived Health and Distresses in Different States

**Figure 1:**
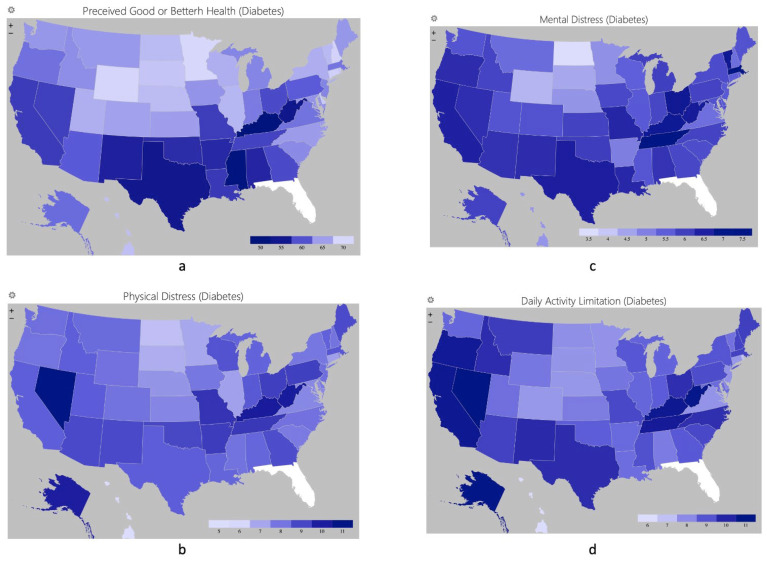
Percentage of perceived good or better health status (**a**), mean days of perceived physical distress (**b**), mental distress (**c**), and daily activity limitation (**d**) among people with diabetes.

**Figure 2:**
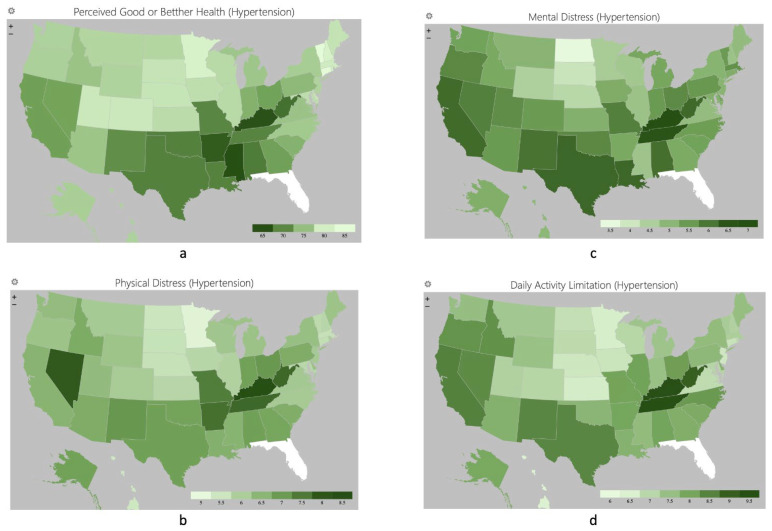
Percentage of perceived good or better health status (**a**), mean days of perceived physical distress (**b**), mental distress (**c**), and daily activity limitation (**d**) among people with hypertension.

The distributions of perceived health and distresses among people with diabetes and hypertension were similar (Figure 1 and Figure 2). People with diabetes and hypertension in the Midwest region were perceived to have the best health statuses and least distresses, while those residing in the Southwest region had the worst perceived health statuses. Attention was required in California, Nevada, Kentucky, and West Virginia, since people with diabetes and hypertension perceived poorer health statuses and more distresses than other states.

### 3.2. Factors Associated with Perceived Health and Distress

For people with diabetes aged 35–64 years (OR = 0.61–0.72), lower education levels (OR = 0.47–0.79), and household incomes of less than 100,000 (OR = 0.22–0.65) were significant factors in reducing the likelihood of perceived good or better health (Table 2). These factors increased their physical distress significantly. With the exception of lower household income, men (Beta = −1.30) and aging (Beta = −9.09–−1.40) were factors with significantly reduced mental distress among people with diabetes. Compared to those retired or unable to work, people with diabetes and able to work perceived a better health status (OR = 1.40–2.22) with reduced distresses (physical: Beta = −5.04–−2.41, mental: Beta = −2.44–−0.60) and daily activity limitations (Beta = −6.34–−2.06).

Table 3 shows the factors associated with health status among people with hypertension. Men (OR = 0.90) aged 25–64 years (OR = 0.48–0.71) with lower education levels (OR = 0.42–0.76) and a household income less than 200,000 (OR = 0.15–0.78) had significantly reduced odds of perceived good or better health. People with hypertension and aged 75 years and above reported less physical distress and daily activity limitations than young adults. Men (Beta = −1.15) and aging (Beta = −8.12–−1.70) were alleviating factors, while lower household income increased the mental distress among people with hypertension (Beta = 0.30–5.48). Those aged 65 years and above (Beta = −3.03–−2.30) and able to work (Beta = −5.51–−2.27) reported less daily activity limitations. No adjustment was made for the regression analyses.

## 4. Discussion

This study assessed the factors associated with perceived health, distresses, and limitations at the national level using 2021 BRFSS survey data. People with diabetes and hypertension were reported to perceive less good health and more distresses and limitations than the general population. Geographic variations were noted, with more residents in the Southwest region (and some states) reporting poorer health and more physical and mental distresses than other regions and states. In the regression analysis, education level, employment status, and household income were the factors associated most with perceived poor health, distresses, and limitations.

A previous national study showed that people with diabetes were the least likely to practice a healthy lifestyle [10], with this study identifying the association of sociodemographic variables on perceived health. Future interventions can target these variables to improve their perceived health, aiming to increase the practice of healthy lifestyle and eventually reduce the risk of developing complications [19]. The interventions may be suitable for patients with hypertension, as the associated sociodemographic variables on perceived health were similar to those with diabetes.

A special pattern was noted when people with diabetes and hypertension became older. Compared to those aged 18–24 years, people with diabetes and hypertension reported having the most physical distress and daily activity limitations at age 45–54 years (Table 2 and Table 3); however, their physical distress and daily activity limitations were improved when they were aged 65 years and older. The pattern might indicate that the needs of older adults were addressed appropriately at a community level. In 2018, the WHO reported the achievements and outlined the strategies to promote age-friendly cities and communities [20]. A recent review of 27 studies found that the age-friendly community interventions were mainly focused on the improvement of physical and mental health [21]; therefore, our analysis found that older adults with diabetes and hypertension reported less physical and mental distresses than young adults.

Our findings showed that the mean day of having mental distress was similar between the general population and people with hypertension, while those with diabetes were reported to have more mental distress. The linear regression showed that people of an older age reported less mental distress. Some interventions, such as acceptance-based interventions, were reviewed to effectively improve the mental distress among people with diabetes, but most of the participants of the included studies in the reviews were aged 50 years and older [22,23]. The mental distress among young adults with diabetes was concerning, and further studies are suggested to ascertain if the interventions are also effective for them.

Employment status and household income were essential factors in health status among people with diabetes and hypertension. In this study, people who were employed would perceive good or better health and less physical and mental distresses than those unemployed. The findings were in line with a previous study indicating that employment status influenced the perceived health status [24]. Although employed people were perceived to have better health, they were less likely to practice healthy lifestyles, resulting in an increased risk of physical health problems [10], and so workplace-based interventions are suggested. Zhou et al. used a propensity score matching technique to examine the effectiveness of a workplace-based hypertension management program among 12,240 people with hypertension [25]. Participants in the intervention group showed a decreased systolic blood pressure during follow-ups in the 2nd, 4th, 6th, 8th, and 10th years, and the control group showed a decrease only in the 2nd and 4th years. A systematic review of 24 studies showed that workplace-based interventions could significantly reduce body weight [26]. On the other hand, this study found that 67.1% and 60.1% of people with diabetes and hypertension were not working, respectively, indicating that this might be a reason for the low household incomes. In turn, people with low household incomes might spend more time increasing their income by working, paying less attention to their physical health conditions, eventually perceiving a poor health status and more physical and mental distresses.

Since the data collection of this study was conducted during the COVID-19 pandemic, the findings of high physical distress might support that people with diabetes and hypertension were associated with poor outcomes in COVID-19 infection [27]. In turn, the high physical distress might also indicate the outcomes of long COVID among such individuals [28]. With the broad range of symptoms of long COVID, people with diabetes and hypertension might require specific supports that are different from other populations.

### 4.1. Implications

Our study findings have strong implications for clinical practice, research, and policy making. Geographic variations in perceived health and distresses highlight the need for reviewing current practices at the state level. Although the questions asked in the BRFSS survey to assess physical distress and mental distress are general, the findings of this study provide insights for future studies that may utilize more specific assessments to investigate the physical and psychological needs among people with diabetes and hypertension. On the other hand, uneven distribution of primary care workforces between states could hinder the health promotion for those with chronic health conditions, such as diabetes and hypertension [29,30]. With the rapid increase in smartphone ownership [31], digital interventions, such as webpages and mobile applications, can be integrated into current practice to reduce both physical and mental distresses [32,33]. Also, the use of digital interventions can reduce the influence of uneven distribution of primary care workforces in healthcare services. However, the government should develop strategies to reduce the difference of uneven distribution of primary care workforces between states, and increased training and payment of locum relief may help in this regard [34].

People with diabetes and hypertension aged 45–54 years reported the most physical distress and daily activity limitations; further studies are suggested to investigate their needs and develop specific interventions that reduce physical distress among such individuals. Physical distress caused by long COVID among people with diabetes and hypertension requires further investigation to develop appropriate interventions. On the other hand, we found that young adults with diabetes and hypertension experienced more mental distress. A future study is suggested to explore their source of stressors, such as newly diagnosed health conditions and workplace−family issues, to develop appropriate interventions for them. For example, daily diabetes management may increase mental distress among young adults with diabetes, and so an educational program with psychological components could reduce their mental distress and improve their glycemic control [35].

Although the unemployed rate among people with diabetes and hypertension was lower than that of the general population in this study, an employed status could improve their physical and mental health; therefore, the government needs to take action to reduce the unemployment rates. Tax deduction can be considered to reinforce the companies to provide regular health promotion activities for their employees. On the other hand, people with low household incomes reported poorer health and more distresses, and barriers to health care access might be a possible reason [36]. Notably, 12.6% of adults did not have health insurance in 2021 [37], and the government can engage with non-governmental organizations to increase health insurance coverage [38]. Employment and health insurance coverage might reduce health expenditures caused by complications with hypertension and diabetes [3,4].

### 4.2. Limitations

The variables in the secondary analysis were limited to the data set used. For example, the majority of people with diabetes and hypertension were older adults, and statistical adjustments to their age in regard to outcome variables were not feasible, as the age is provided in categories in the dataset. All variables were reported by individuals via phone call, which might lead to a reporting bias arising from recollections coming from memory. Data collection was conducted during the COVID-19 pandemic, which may have influenced the respondents of this survey; however, the use of a national data set enhanced the generalizability of our findings.

## 5. Conclusions

The analysis of the national data set indicated that people with chronic illness were perceived to have poorer health statuses and more physical and mental distresses than the general population, especially those with diabetes. Geographic variations in perceived health and distresses were noted. More attention is suggested to be given to the people with diabetes and hypertension living in the Southwest region. Through regression analysis, some sociodemographic variables, such as age and education level, were identified as significant factors influencing their perceived health and distress. In addition to physical health conditions, several interventions were suggested to strengthen current practice, while policy makers have important roles in addressing several influencing variables.

## Figures and Tables

**Table 1:** Individual characteristics and health status.

	All	Diabetes	Hypertension
	N = 333,316	N = 43,911	N = 130,960
Sex			
Male	159,355 (47.8%)	22,400 (51.0%)	66,956 (51.1%)
Female	173,961 (52.2%)	21,511 (49.0%)	64,004 (48.9%)
Age range (years)			
18 to 24	17,124 (5.1%)	198 (0.5%)	1343 (1.0%)
25 to 34	38,404 (11.6%)	820 (1.9%)	4825 (3.7%)
35 to 44	48,309 (14.5%)	2466 (5.6%)	10,172 (7.8%)
45 to 54	51,345 (15.4%)	5704 (13.0%)	17,084 (13.0%)
55 to 64	65,719 (19.7%)	11,186 (25.4%)	30,378 (23.2%)
65 to 74	67,757 (20.3%)	14,141 (32.2%)	38,704 (29.6%)
75 up	44,658 (13.4%)	9396 (21.4%)	28,454 (21.7%)
Residential area			
Urban	284,944 (85.5%)	36,604 (83.4%)	109,565 (83.7%)
Rural	48,372 (14.5%)	7307 (16.6%)	21,395 (16.3%)
Education level			
Attended high school or less	17,652 (5.3%)	3551 (8.1%)	7930 (6.1%)
Graduated from high school	81,692 (24.5%)	12,715 (29.0%)	35,861 (27.4%)
Attended college or above	93,203 (28.0%)	13,622 (31.0%)	38,598 (29.5%)
Graduated from college or above	140,769 (42.2%)	14,023 (31.9%)	48,571 (37.0%)
Employment status			
Employed	182,528 (54.7%)	14,453 (32.9%)	52,282 (39.9%)
Unemployed	34,854 (10.5%)	3320 (7.6%)	9778 (7.5%)
Retired or unable to work	115,934 (34.8%)	26,138 (59.5%)	68,900 (52.6%)
Household income (US dollars)			
Less than 15,000	20,616 (6.2%)	4580 (10.4%)	10,088 (7.7%)
15,000 to <25,000	34,237 (10.3%)	6825 (15.5%)	16,730 (12.8%)
25,000 to <35,000	42,321 (12.7%)	7040 (16.0%)	18,492 (14.2%)
35,000 to <50,000	46,834 (14.1%)	6919 (15.8%)	20,089 (15.3%)
50,000 to <100,000	104,756 (31.4%)	12,077 (27.5%)	39,664 (30.3%)
100,000 to <200,000	66,017 (19.8%)	5430 (12.4%)	20,995 (16.0%)
200,000 or more	18,535 (5.5%)	1040 (2.4%)	4902 (3.7%)
Perceived health			
Excellent	58,798 (17.6%)	1656 (3.8%)	10,722 (8.2%)
Very good	116,103 (34.8%)	8400 (19.1%)	38,373 (29.3%)
Good	103,882 (31.2%)	17,086 (38.9%)	48,439 (37.0%)
Fair	40,593 (12.2%)	11,867 (27.0%)	24,242 (18.5%)
Poor	13,940 (4.2%)	4902 (11.2%)	9184 (7.0%)
Perceived unhealthy day, mean (SD)			
Physical distress	3.80 (8.32)	7.25 (11.05)	5.37 (9.79)
Mental distress	4.23 (8.20)	4.79 (9.05)	4.28 (8.52)
Daily activity limitation	5.18 (8.94)	7.89 (10.96)	6.52 (10.12)

**Table 2:** Factors associated with health status among people with diabetes.

	Perceived Good or Better Health ^a^	Physical Distress ^b^	Mental Distress ^b^	Daily Activity Limitation ^b^
	Odds Ratio (95% CI)	Beta (95% CI)	Beta (95% CI)	Beta (95% CI)
Sex: male	0.97 (0.93, 1.01)	0.01 (−0.19, 0.22)	−1.30 (−1.47, −1.14) *	0.69 (0.43, 0.95) *
Age range (years)				
18 to 24	Reference	Reference	Reference	Reference
25 to 34	0.78	1.14	−1.40	0.95
	(0.55, 1.10)	(−0.51, 2.80)	(−2.75, −0.06) *	(−0.88, 2.78)
35 to 44	0.61	2.83	−2.27	1.10
	(0.44, 0.85) *	(1.28, 4.37) *	(−3.52, −1.01) *	(−0.60, 2.81)
45 to 54	0.63	3.27	−3.47	1.14
	(0.46, 0.87) *	(1.76, 4.78) *	(−4.70, −2.24) *	(−0.53, 2.80)
55 to 64	0.72	3.00	−5.09	0.74
	(0.52, 0.98) *	(1.50, 4.50) *	(−6.31, −3.87) *	(−0.91, 2.40)
65 to 74	1.13	0.07	−7.93	−2.29
	(0.82, 1.69)	(−1.44, 1.57)	(−9.16, −6.71) *	(−3.95, −0.63)
75 up	1.23	−0.36	−9.09	−2.99
	(0.89, 1.69)	(−1.88, 1.15)	(−10.33, −7.86) *	(−4.67, −1.32)
Residence in urban areas	1.06	−0.09	0.29	−0.20
	(1.01, 1.12) *	(−0.36, 0.18)	(0.07, 0.50) *	(−0.54, 0.14)
Education level				
Attended high school or less	0.47	0.87	−0.22	0.54
	(0.43, 0.51) *	(0.44, 1.30) *	(−0.57, 0.12)	(0.02, 1.06) *
Graduated from high school	0.73	0.46	−0.24	0.23
	(0.69, 0.77) *	(0.19, 0.74) *	(−0.47, −0.02) *	(−0.12, 0.59)
Attended college or above	0.79	0.76	0.34	0.42
	(0.75, 0.84) *	(0.50, 1.02) *	(0.13, 0.55) *	(0.08, 0.75) *
Graduated from college or above	Reference	Reference	Reference	Reference
Employment status				
Employed	2.22	−5.04	−2.44	−6.34
	(2.09, 2.35) *	(−5.31, −4.76) *	(−2.66, −2.21) *	(−6.69, −5.99) *
Unemployed	1.40	−2.41	−0.60	−2.06
	(1.30, 1.52) *	(−2.82, −2.01) *	(−0.93, −0.27) *	(−2.54, −1.58) *
Retired or unable to work	Reference	Reference	Reference	Reference
Household income (US dollars)				
Less than 15,000	0.22	6.21	4.84	4.78
	(0.19, 0.27) *	(5.46, 6.97) *	(4.22, 5.46) *	(3.72, 5.83) *
15,000 to <25,000	0.29	4.47	2.97	3.15
	(0.25, 0.35) *	(3.74, 5.19) *	(2.37, 3.56) *	(2.12, 4.18) *
25,000 to <35,000	0.37	3.15	2.01	2.08
	(0.31, 0.44) *	(2.43, 3.86) *	(1.43, 2.60) *	(1.06, 3.10) *
35,000 to <50,000	0.46	1.95	1.50	1.36
	(0.39, 0.55) *	(1.25, 2.66) *	(0.92, 2.08) *	(0.34, 2.38) *
50,000 to <100,000	0.65	1.07	0.61	0.54
	(0.54, 0.77) *	(0.39, 1.75) *	(0.06, 1.17) *	(−0.45, 1.53)
100,000 to <200,000	0.86	0.07	0.01	0.21
	(0.71, 1.02)	(−0.64, 0.77)	(−0.56, 0.59)	(−0.82, 1.24)
200,000 or more	Reference	Reference	Reference	Reference

^a^ Logistic regression. ^b^ Linear regression. * Statistical significance, *p* < 0.05.

**Table 3:** Factors associated with health status among people with hypertension.

	Perceived Good or Better Health ^a^	Physical Distress ^b^	Mental Distress ^b^	Daily Activity Limitation ^b^
	Odds Ratio (95% CI)	Beta (95% CI)	Beta (95% CI)	Beta (95% CI)
Sex: male	0.97	0.01	−1.30	0.69
	(0.93, 1.01)	(−0.19, 0.22)	(−1.47, −1.14) *	(0.43, 0.95) *
Age range (years)				
18 to 24	Reference	Reference	Reference	Reference
25 to 34	0.78	1.14	−1.40	0.95
	(0.55, 1.10)	(−0.51, 2.80)	(−2.75, −0.06) *	(−0.88, 2.78)
35 to 44	0.61	2.83	−2.27	1.10
	(0.44, 0.85) *	(1.28, 4.37) *	(−3.52, −1.01) *	(−0.60, 2.81)
45 to 54	0.63	3.27	−3.47	1.14
	(0.46, 0.87) *	(1.76, 4.78) *	(−4.70, −2.24) *	(−0.53, 2.80)
55 to 64	0.72	3.00	−5.09	0.74
	(0.52, 0.98) *	(1.50, 4.50) *	(−6.31, −3.87) *	(−0.91, 2.40)
65 to 74	1.13	0.07	−7.93	−2.29
	(0.82, 1.69)	(−1.44, 1.57)	(−9.16, −6.71) *	(−3.95, −0.63) *
75 up	1.23	−0.36	−9.09	−2.99
	(0.89, 1.69)	(−1.88, 1.15)	(−10.33, −7.86) *	(−4.67, −1.32) *
Residence in urban areas	1.06	−0.09	0.29	−0.20
	(1.01, 1.12) *	(−0.36, 0.18)	(0.07, 0.50) *	(−0.54, 0.14)
Education level				
Attended high school or less	0.47	0.87	−0.22	0.54
	(0.43, 0.51) *	(0.44, 1.30) *	(−0.57, 0.12)	(0.02, 1.06) *
Graduated from high school	0.73	0.46	−0.24	0.23
	(0.69, 0.77) *	(0.19, 0.74) *	(−0.47, −0.02) *	(−0.12, 0.59)
Attended college or above	0.79	0.76	0.34	0.42
	(0.75, 0.84) *	(0.50, 1.02) *	(0.13, 0.55) *	(0.08, 0.75) *
Graduated from college or above	Reference	Reference	Reference	Reference
Employment status				
Employed	2.22	−5.04	−2.44	−6.34
	(2.09, 2.35) *	(−5.31, −4.76) *	(−2.66, −2.21) *	(−6.69, −5.99) *
Unemployed	1.40	−2.41	−0.60	−2.06
	(1.30, 1.52) *	(−2.82, −2.01) *	(−0.93, −0.27) *	(−2.54, −1.58) *
Retired or unable to work	Reference	Reference	Reference	Reference
Household income (US dollars)				
Less than 15,000	0.22	6.21	4.84	4.78
	(0.19, 0.27) *	(5.46, 6.97) *	(4.22, 5.46) *	(3.72, 5.83) *
15,000 to <25,000	0.29	4.47	2.97	3.15
	(0.25, 0.35) *	(3.74, 5.19) *	(2.37, 3.56) *	(2.12, 4.18) *
25,000 to <35,000	0.37	3.15	2.01	2.08
	(0.31, 0.44) *	(2.43, 3.86) *	(1.43, 2.60) *	(1.06, 3.10) *
35,000 to <50,000	0.46	1.95	1.50	1.36
	(0.39, 0.55) *	(1.25, 2.66) *	(0.92, 2.08) *	(0.34, 2.38) *
50,000 to <100,000	0.65	1.07	0.61	0.54
	(0.54, 0.77) *	(0.39, 1.75) *	(0.06, 1.17) *	(−0.45, 1.53)
100,000 to <200,000	0.86	0.07	0.01	0.21
	(0.71, 1.02)	(22120.64, 0.77)	(−0.56, 0.59)	(−0.82, 1.24)
200,000 or more	Reference	Reference	Reference	Reference

^a^ Logistic regression. ^b^ Linear regression. * Statistical significance, *p* < 0.05.

## Data Availability

The data used in this study (Behavioral Risk Factor Surveillance System 2021) are publicly available [18].

## References

[1] WHO The Top 10 Causes of Death. https://www.who.int/news-room/fact-sheets/detail/the-top-10-causes-of-death.

[2] GBD 2019 Risk Factors Collaborators (2020). Global burden of 87 risk factors in 204 countries and territories, 1990–2019: A systematic analysis for the Global Burden of Disease Study 2019. Lancet.

[3] Wierzejska E., Giernas B., Lipiak A., Karasiewicz M., Cofta M., Staszewski R. (2020). A global perspective on the costs of hypertension: A systematic review. Arch. Med. Sci..

[4] Seuring T., Archangelidi O., Suhrcke M. (2015). The Economic Costs of Type 2 Diabetes: A Global Systematic Review. Pharmacoeconomics.

[5] Unger T., Borghi C., Charchar F., Khan N.A., Poulter N.R., Prabhakaran D., Ramirez A., Schlaich M., Stergiou G.S., Tomaszewski M. (2020). 2020 International Society of Hypertension Global Hypertension Practice Guidelines. Hypertension.

[6] Williams B., Mancia G., Spiering W., Agabiti Rosei E., Azizi M., Burnier M., Clement D.L., Coca A., de Simone G., Dominiczak A. (2018). 2018 ESC/ESH Guidelines for the management of arterial hypertension. Eur. Heart J..

[7] American Diabetes Association (2022). Standards of Medical Care in Diabetes—2022 Abridged for Primary Care Providers. Clin. Diabetes.

[8] de Boer I.H., Bangalore S., Benetos A., Davis A.M., Michos E.D., Muntner P., Rossing P., Zoungas S., Bakris G. (2017). Diabetes and Hypertension: A Position Statement by the American Diabetes Association. Diabetes Care.

[9] WHO Noncommunicable Diseases. https://www.who.int/news-room/fact-sheets/detail/noncommunicable-diseases.

[10] Tam H.L., Chair S.Y., Leung I.S.H., Leung L.Y.L., Chan A.S.W. (2023). US Adults Practicing Healthy Lifestyles before and during COVID-19: Comparative Analysis of National Surveys. JMIR Public Health Surveill..

[11] Axon D.R., Jang A., Son L., Pham T. (2022). Determining the association of perceived health status among united states older adults with self-reported pain. Aging Health Res..

[12] Tinajero-Chávez L.I., Mora-Romo J.F., Bravo-Doddoli A., Cruz-Narciso B.V., Calleja N., Toledano-Toledano F. (2023). Design, Development, and Validation of the Self-Perceived Health Scale (SPHS). Healthcare.

[13] Perrin N.E., Davies M.J., Robertson N., Snoek F.J., Khunti K. (2017). The prevalence of diabetes-specific emotional distress in people with Type 2 diabetes: A systematic review and meta-analysis. Diabet. Med..

[14] Young-Hyman D., de Groot M., Hill-Briggs F., Gonzalez J.S., Hood K., Peyrot M. (2016). Psychosocial Care for People with Diabetes: A Position Statement of the American Diabetes Association. Diabetes Care.

[15] Ojike N., Sowers J.R., Seixas A., Ravenell J., Rodriguez-Figueroa G., Awadallah M., Zizi F., Jean-Louis G., Ogedegbe O., McFarlane S.I. (2016). Psychological Distress and Hypertension: Results from the National Health Interview Survey for 2004–2013. Cardiorenal Med..

[16] Duenas M., Salazar A., de Sola H., Failde I. (2020). Limitations in Activities of Daily Living in People with Chronic Pain: Identification of Groups Using Clusters Analysis. Pain. Pract..

[17] von Elm E., Altman D.G., Egger M., Pocock S.J., Gotzsche P.C., Vandenbroucke J.P., STORBE Initiative (2007). The Strengthening the Reporting of Observational Studies in Epidemiology (STROBE) statement: Guidelines for reporting observational studies. Prev. Med..

[18] 2021 BRFSS Survey Data and Documentation. https://www.cdc.gov/brfss/annual_data/annual_2021.html.

[19] Li Y., Schoufour J., Wang D.D., Dhana K., Pan A., Liu X., Song M., Liu G., Shin H.J., Sun Q. (2020). Healthy lifestyle and life expectancy free of cancer, cardiovascular disease, and type 2 diabetes: Prospective cohort study. BMJ.

[20] WHO The Global Network for Age-Friendly Cities and Communities. https://www.who.int/publications/i/item/WHO-FWC-ALC-18.4.

[21] Hong A., Welch-Stockton J., Kim J.Y., Canham S.L., Greer V., Sorweid M. (2023). Age-Friendly Community Interventions for Health and Social Outcomes: A Scoping Review. Int. J. Environ. Res. Public. Health.

[22] Schmidt C.B., van Loon B.J.P., Vergouwen A.C.M., Snoek F.J., Honig A. (2018). Systematic review and meta-analysis of psychological interventions in people with diabetes and elevated diabetes-distress. Diabet. Med..

[23] Ngan H.Y., Chong Y.Y., Chien W.T. (2021). Effects of mindfulness- and acceptance-based interventions on diabetes distress and glycaemic level in people with type 2 diabetes: Systematic review and meta-analysis. Diabet. Med..

[24] Park S., Chan K.C., Williams E.C. (2016). Gain of employment and perceived health status among previously unemployed persons: Evidence from a longitudinal study in the United States. Public. Health.

[25] Zhou Y.F., Chen S., Wang G., Chen S., Zhang Y.B., Chen J.X., Tu Z.Z., Liu G., Wu S., Pan A. (2022). Effectiveness of a Workplace-Based, Multicomponent Hypertension Management Program in Real-World Practice: A Propensity-Matched Analysis. Hypertension.

[26] Mulchandani R., Chandrasekaran A.M., Shivashankar R., Kondal D., Agrawal A., Panniyammakal J., Tandon N., Prabhakaran D., Sharma M., Goenka S. (2019). Effect of workplace physical activity interventions on the cardio-metabolic health of working adults: Systematic review and meta-analysis. Int. J. Behav. Nutr. Phys. Act..

[27] Hosseinzadeh R., Goharrizi M., Bahardoust M., Alvanegh A.G., Ataee M.R., Bagheri M., Navidiyan E.S., Zijoud S.R.H., Heiat M. (2021). Should all patients with hypertension be worried about developing severe coronavirus disease 2019 (COVID-19)?. Clin. Hypertens..

[28] Davis H.E., McCorkell L., Vogel J.M., Topol E.J. (2023). Long COVID: Major findings, mechanisms and recommendations. Nat. Rev. Microbiol..

[29] Zhang D., Son H., Shen Y., Chen Z., Rajbhandari-Thapa J., Li Y., Eom H., Bu D., Mu L., Li G. (2020). Assessment of Changes in Rural and Urban Primary Care Workforce in the United States from 2009 to 2017. JAMA Netw. Open.

[30] Tam H.L., Chung S.F., Wang Q. (2023). Urban-rural disparities in hypertension management among middle-aged and older patients: Results of a 2018 Chinese national study. Chronic Illn..

[31] Pew Research Center Smartphone Ownership Is Growing Rapidly around the World, but Not Always Equally. https://www.pewresearch.org/global/2019/02/05/smartphone-ownership-is-growing-rapidly-around-the-world-but-not-always-equally.

[32] Babbage C.M., Jackson G.M., Davies E.B., Nixon E. (2022). Self-help Digital Interventions Targeted at Improving Psychological Well-being in Young People with Perceived or Clinically Diagnosed Reduced Well-being: Systematic Review. JMIR Ment. Health.

[33] Nicholl B.I., Sandal L.F., Stochkendahl M.J., McCallum M., Suresh N., Vasseljen O., Hartvigsen J., Mork P.J., Kjaer P., Søgaard K. (2017). Digital Support Interventions for the Self-Management of Low Back Pain: A Systematic Review. J. Med. Internet Res..

[34] Russell D., Mathew S., Fitts M., Liddle Z., Murakami-Gold L., Campbell N., Ramjan M., Zhao Y., Hines S., Humphreys J.S. (2021). Interventions for health workforce retention in rural and remote areas: A systematic review. Hum. Resour. Health.

[35] Ngan H.Y., Chong Y.Y., Loo K.M., Chien W.T. (2023). Preliminary efficacy of an acceptance-based diabetes education (ACT-DE) programme for people with type 2 diabetes on diabetes distress and self-care behaviours: A pilot randomised controlled trial. J. Context. Behav..

[36] Lazar M., Davenport L. (2018). Barriers to Health Care Access for Low Income Families: A Review of Literature. J. Community Health Nurs..

[37] Terlizzi E.P., Cohen R.A. (2022). Geographic Variation in Health Insurance Coverage: United States, 2021. Natl. Health Stat. Rep..

[38] Sanadgol A., Doshmangir L., Majdzadeh R., Gordeev V.S. (2021). Engagement of non-governmental organisations in moving towards universal health coverage: A scoping review. Glob. Health.

